# Ageing reveals the latent effects of early life stress on respiratory and metabolic function in female rats: Novel insights into the sex‐specific origins of sleep apnoea

**DOI:** 10.1113/EP092722

**Published:** 2025-07-20

**Authors:** Danuzia Ambrozio‐Marques, Loralie Mei Guay, Alicia A. Koogler, Tim D. Ostrowski, Richard Kinkead

**Affiliations:** ^1^ Research Center of the Québec Heart & Lung Institute Québec Canada; ^2^ Department of Pediatrics, Faculty of Medicine Université Laval Québec Canada; ^3^ Department of Physiology A.T. Still University Kirksville Missouri USA

**Keywords:** ageing, control of breathing, leptin, obesity, stress

## Abstract

Sleep apnoea (SA) is ∼2 times more prevalent in men than women. However, this changes at menopause as the occurrence of SA increases and matches that of men. Menopause is a natural process, but it remains unclear why SA emerges only in a subpopulation of ageing women. Early life stress has persistent and sex‐specific effects on the brain. Since loss of ovarian hormones is commonly invoked to explain the emergence of various diseases in menopausal women, we tested the hypothesis that the impacts of early life stress on respiratory control remain latent until they reach old age. Newborn rats were either subjected to neonatal maternal separation (NMS; 3 h/day; postnatal days 3–12) or remained undisturbed (CTRL). Females were then raised under standard conditions until they reached adulthood (12–17 weeks), middle‐age (35–40 weeks) or old age (60–65 weeks). Respiratory activity was measured with whole body plethysmography. Body weight and composition was assessed with nuclear magnetic resonance. NMS augmented the apnoea index; however, this effect was detected only in old females. Body mass index and the percentage of body fat increased progressively; these changes were enhanced by NMS and most notable in old females. We conclude that NMS compromises the ageing trajectory of female rats and leads to the development of a phenotype that shares many features reported in SA patients, including respiratory and metabolic dysfunction. Prior life experiences may be an important risk factor in the development of SA in ageing women.

## Abstract (highlights)

- **What is the central question of this study?**
  In women, the prevalence of sleep apnoea rises significantly at menopause, but not all women develop this respiratory problem: does prior exposure to stress influences the ageing trajectory in female rats?
- **What is the main finding and its importance?**
  By comparison with unstressed rats, 1‐year‐old females previously exposed to neonatal maternal separation develop a phenotype that shares many features reported in SA patients, including a greater number of apnoeas, exaggerated CO_2_ chemoreflex and metabolic dysfunction. Prior life experiences may be an important risk factor in the development of SA in ageing women.

## 1 INTRODUCTION

The proportion of older people is growing rapidly and it is estimated that by 2050, more than one‐fifth of the global population will be 60 years or older. Identifying the factors that increase the risk of disease is essential to attenuate the burden of chronic health issues for families and society. Sleep is an important determinant of health and is fundamental to sustain brain functions during ageing (Lewis, 2021). Old age is associated with longer sleep latency, reduced sleep quality and more fragile sleep (García‐García et al., 2023). Sleep apnoea (SA) is an important, yet often underestimated respiratory disease that disrupts sleep; SA affects nearly 1 billion adults worldwide and consists of decreases or complete cessations of airflow to the lungs (hypopneas or apnoeas, respectively) during sleep (Benjafield et al., 2019). Apnoeas commonly lead to recurrent drops in arterial O_2_ level that stimulate the sympathetic system, the stress pathways, and ultimately promote inflammation (Lavie, 2015). Because apnoeic events are often resolved by arousals, sleep fragmentation and daytime sleepiness are significant consequences of SA. With time, sleep disruption and recurrent hypoxaemias are deleterious to healthy ageing as it increases the risk of various diseases, including hypertension, depression and obesity (Osorio et al., 2022).

The pathophysiology of SA is complex and multifactorial, yet obstruction of the upper airways and unstable neural control of breathing are important causes of SA (Eckert, 2018). Obesity is an aggravating factor as deposition of adipose tissue in the neck area favours airway collapse and hypoventilation during sleep (Pham et al., 2022). Consequently, the body mass index (BMI) is positively correlated with the severity of SA. Furthermore, the prevalence and manifestations of SA show significant sex‐based differences (Kinkead et al., 2021). Owing to an occurrence ∼2 times greater in men than woman, SA has been long considered ‘a man's disease’ such that women reporting sleep problems have often been misdiagnosed. This is an important issue because female SA patients report sleep‐related problems more frequently than men (Dancey et al., 2001; Kinkead et al., 2021). Notwithstanding, it is now established that the incidence of SA rises at menopause and attains an occurrence comparable to men, and while the BMI often increases at menopause, the severity of SA between pre‐ and post‐menopause persists after adjusting for BMI and neck circumference (Heinzer et al., 2018; White & Younes, 2012). Thus, by comparison with men, the causes of SA in post‐menopausal women may reside more in respiratory control anomalies, than anatomical differences in the upper airways (Conde et al., 2023). However, this remains to be tested experimentally.

In women, the factors responsible for the age‐related rise in SA are not well known, but progressive loss of the ‘protective effects’ of ovarian hormones is commonly invoked to explain the emergence of cardiovascular diseases in menopausal women (Conde et al., 2023; Kinkead et al., 2021; Netzer et al., 2003). Menopause is a natural process, but it remains unclear why SA emerges only in a subpopulation of ageing women. Elucidating the aetiology of SA in women is key to proper treatment, yet our comprehension of the different ageing trajectories in women remains limited.

Stress experienced during early life is a major cause of adult disease that can ultimately compromise health at various life stages (Heim et al., 2019; Shonkoff et al., 2009). Stress has persistent and sex‐specific effects on the brain and although disruption of the neural system regulating breathing is an important pathophysiological trait in SA, the contribution of stress in the age‐dependent emergence of this respiratory disorder in women is yet to be addressed. These principles raise the possibility that in women, the deleterious impacts of early life stress on respiratory control remain latent until they reach menopause. To address this idea experimentally, we tested the hypothesis that neonatal maternal separation (NMS), a well established and clinically relevant animal model of early life stress, exacerbates the changes in respiratory control associated with natural ageing. To do so, we compared the ageing trajectory of control female rats raised under standard animal care to that of females subjected to NMS (3 h/day from postnatal day 3 to 12). Quantification of the apnoea index during sleep and respiratory reflexes were performed at three distinct age groups: young adults (∼8 weeks), ‘middle‐age’ (∼40 weeks) and old (∼60 weeks). We explored the potential contribution of obesity to age‐related change in respiratory control by assessing body composition and leptin and cytokine signalling. Immunohistochemistry studies were performed on selected brain regions to evaluate how NMS and ageing affect the basal activity and cell structure of this essential component of the respiratory control network. Although the present study focused exclusively on female rats to investigate the interaction between reproductive ageing and respiratory control, previous work from our group demonstrated that early life stress also induces lasting respiratory alterations in male rats (Kinkead et al., 2009).

## 2 METHODS

### 2.1 Ethical approval and animals

All protocols were conducted in accordance with guidelines outlined by the Canadian Council on Animal Care and approved by the Laval University Animal Care Committee (protocol no. 2020‐441). Descriptions of the key information regarding animal use and experimental protocols are in line with the ARRIVE guidelines 2.0. and the guidelines of the Canadian Council on Animal Care. All personnel involved in this research followed appropriate training and complied with the institutional and national ethical guidelines, which are consistent with the standards of *Experimental Physiology*. Except for rats used for reproduction, all experimental animals were born and raised in the animal care facilities of the Québec Heart and Lung Institute Research Centre. Adult male and nulliparous female Sprague–Dawley rats used for reproduction were sourced from Charles River Laboratories (Montreal, Canada). Animals were housed in pairs under a 12‐h light–dark cycle with food and water available ad libitum.

### 2.2 Experimental model, and overview of study design

Following mating, gestating females were housed individually. Large litters were reduced to 12 pups with an equal sex ratio within 2 days of birth. Each litter was randomly assigned to either the neonatal maternal separation (NMS) protocol or left undisturbed (Control, CTRL) according to established protocols (Genest et al., 2007; Tenorio‐Lopes et al., 2017). Briefly, pups subjected to NMS were placed in a temperature‐ and humidity‐controlled incubator (33°C, 45% relative humidity) for 3 h/day (09.00 to 12.00 h) from postnatal day 3 to 12. Animals were weaned on postnatal day 21 and subsequently housed in pairs until reaching adulthood. Note that only females were used in this study; males were assigned to other projects performed by our team or collaborators. Each experimental group was composed of rats originating from at least three different litters to reduce the likelihood of confounding effects of litter‐specific differences on treatment outcomes.

As the main goal of the study was to evaluate the impacts of NMS on the ageing trajectory of females, rats were randomly assigned to three distinct age groups reflecting key reproductive phases: (1) young adults: between 12–17 weeks old, representing the phase of full sexual maturity; (2) a middle‐age group: between 35 and 40 weeks old, characterized by the onset of irregularities in oestrous cycles and disrupted gonadotropin secretion (Diaz Brinton, 2012; Nass et al., 1984); and (3) an old age group: between 60 and 65 weeks old, corresponding to reproductive senescence and a menopause‐like state, as detailed in the work of Diaz Brinton (2012). As each animal was subjected to multiple measurements, care was taken to avoid carry‐over effects and interference with previous procedures on the variable of interest. For instance, body composition was measured 1 day after respiratory function; brain and blood were collected 1 week after the last experimental procedure. Following measurements, the oestrous cycle was evaluated as previously described in Marques et al. (2015). Briefly, vaginal smears were collected and immediately transferred onto glass slides, then observed under a microscope at ×10 magnification to determine the stage of the oestrous cycle. Each slide was assessed for the presence of cornified epithelial cells, nucleated epithelial cells and leukocytes to classify the cycle stage using the following criteria: oestrus: predominantly cornified epithelial cells; metoestrus: presence of nucleated cells with a predominance of leukocytes; dioestrus: predominantly leukocytes; and pro‐oestrus: predominantly nucleated epithelial cells. The oestrous cycle was considered only in young females, and we conducted a separate analysis to assess its potential impact on the measured variables. However, there were no significant effects of oestrous cycle on any of our analyses. Since the oestrous cycle did not influence our main findings, we opted to exclude detailed reporting for clarity and conciseness. All experiments were carried out between 08.00 and 13.00 h to attenuate the impact of circadian rhythm on the variables of interest.

### 2.3 Blood collection and brain harvesting

One week after the last measurements were performed, rats were deeply anaesthetized with an intraperitoneal injection of a mixture of ketamine (80 mg kg^−1^) and xylazine (10 mg kg^−1^). Terminal blood samples were withdrawn from the left ventricle; samples were placed in a serum‐gel clotting activator Microtube (Sarstedt AG & Co., Nümbrecht Germany). After centrifugation (10000 g at 4°C for 12 min), blood serum was collected and placed in a −80°C freezer until assayed.

Intra‐cardiac perfusion was then performed with 0.9% saline followed by 4% paraformaldehyde (PFA) in 0.1 M sodium tetraborate buffer (PFA/borax; pH 9.5 at 4°C). Brains were removed from the skull, post‐fixed for 24 h in 4% PFA/borax, and then placed in 30% sucrose–4% paraformaldehyde solution for 48 h at 4°C. After, brains were placed in a −80°C freezer until they were sectioned for immunohistochemistry.

### 2.4 Non‐invasive quantification of respiratory activity at rest and during acute stimulation

Respiratory function was measured at rest (normoxia) and during hypercapnic and hypoxic challenges using a whole‐body plethysmograph (EMKA Technologies, Paris, France). Room air was delivered into the experimental chamber at a constant rate (1.3–1.6 L min^−1^) with a bias flow regulator (PLY1020; Buxco Electronics, Sharon, CT, USA). During acute stimulation, hypoxia and hypercapnia was induced by adding N_2_ or CO_2_, respectively to the inflowing gas (see details below). Respiratory parameters, including frequency, tidal volume and minute ventilation, were recorded and analysed using a data acquisition system (Spike2 software, Cambridge Electronic Design (CED), Cambridge, UK) using established procedures in our group (Joseph et al., 2020). Briefly, respiratory measurements were performed between ∼08.00 and 13.00 h, when rats normally sleep. The plethysmography signal was calibrated by injecting 1 mL of air into the chamber at a rate corresponding to the airflow range typically generated by the rat. Water vapor pressure, CO_2_ and O_2_ levels in the out‐flowing air were continuously monitored using specific gas analysers (RH‐300, CA‐10, and FC‐10, Sable Systems, Las Vegas, NV, USA). The O_2_ and CO_2_ analysers were calibrated using a certified dry gas mix (Linde Canada Limited, Vanier, QC, Canada). Chamber temperature and barometric pressure were measured at the beginning and end of the experiment. These values, along with relative humidity and body temperature of the animals (*T*_B_), were used to express the tidal volume (*V*_T_ and thus minute ventilation, ${\dot{V}}_{E}$) in mL BTPS (Drorbaugh & Fenn, 1955). Composition of the gas mixtures flowing in and out of the chamber was used for subsequent calculation of oxygen consumption (${\dot{V}}_{O_{2}}$) and CO_2_ production (${\dot{V}}_{CO_{2}}$) in an open system (Boukari et al., 2017; Ganouna‐Cohen et al., 2022); further details are provided in the next section. All signals were digitized (Micro 1401 data acquisition system, CED) and stored on a computer running Spike2 software (version 7.06, CED).

**FIGURE A1:**
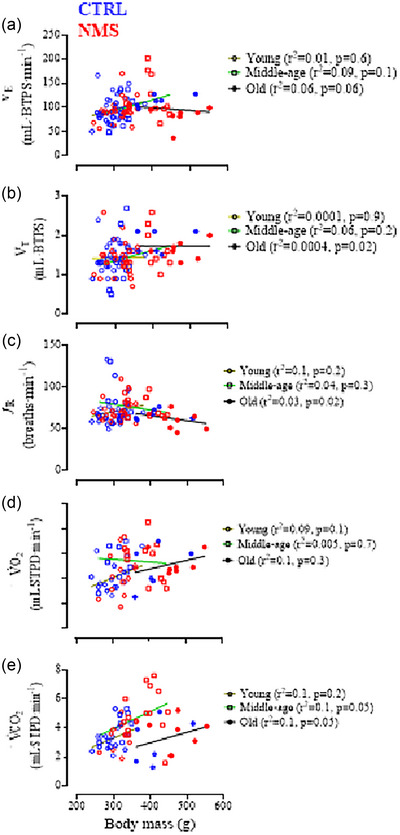
Relationship between body mass and respiratory or metabolic parameters while breathing room air (normoxia). Each dot represents an individual animal, separated by age group: young (open circles), middle‐aged (open squares), and old (filled symbols). (a) Minute ventilation (${\dot{V}}_{E}$, mL BTPS min⁻¹); (b) Tidal volume (*V*_T_, mL BTPS); (c) Breathing frequency (*f*_R_, breaths min⁻¹) and (d) Carbon dioxide production (${\dot{V}}_{CO_{2}}$, mL STPD min⁻¹), presented in function of body mass. Lines represent linear regressions for each age group, with corresponding coefficients of determination (*r* ^2^) and *P*‐values reported in the legend. The CTRL animals are in blue and the NMS in red.

Given that the variability in body mass across age groups is significant and does not scale linearly, we initially applied standard allometric corrections based on the equations proposed by Mortola et al. (1994). However, detailed analysis revealed differences in scaling relationships between CTRL and NMS‐exposed rats, suggesting potential inaccuracies in applying the same correction across treatments (Figure A1 in the Appendix). To address this issue, we performed an analysis of covariance (ANCOVA), which considered body mass as a covariate, with age and stress as fixed factors. The ANCOVA model included interaction terms (mass × age, mass × stress, and mass × age × stress) to comprehensively assess any potential confounding effects or interactions involving body mass (Table 1).

**TABLE 1:** Results of analysis of covariance evaluating the effects of body mass, age and neonatal maternal separation on basal (normoxic) respiratory variables

Dependent variable	Stress effect	Age effect	Mass effect (covariate)	Stress × age	Mass × age	Mass × stress	Mass × age × stress
${\dot{V}}_{E}$ (mL min^−1^)	*F*_(1,117) _= 0.1, *P =* 0.7	*F*_(2,117) _= 0.5, *P =* 0.5	*F*_(1,117) _= 0.007, *P =* 0.9	*F*_(2,117) _= 0.02, *P =* 0.9	*F*_(2,117) _= 0.3, *P =* 0.7	*F*_(1,117) _= 0.1, *P =* 0.7	*F*_(2,117) _= 0.02, *P =* 0.9
*V*_T_ (mL min^−1^)	*F*_(1,117) _= 0.03, *P =* 0.8	*F*_(2,117) _= 0.2, *P =* 0.7	*F*_(1,117) _= 0.001, *P =* 0.9	*F*_(1,117) _= 0.04, *P =* 0.8	*F*_(2,117) _= 0.1, *P =* 0.9	*F*_(2,117) _= 0.2, *P =* 0.8	*F*_(1,117) _= 0.03, *P =* 0.8
*f*_R_ (breaths min^−1^)	*F*_(1,117) _= 0.04, *P =* 0.8	*F*_(2,117) _= 1.4, *P =* 0.2	*F*_(1,117) _= 0.02, *P =* 0.8	*F*_(1,117) _= 0.02, *P =* 0.8	*F*_(2,117) _= 1.1, *P =* 0.3	*F*_(2,117) _= 0.4, *P =* 0.6	*F*_(1,117) _= 0.04, *P =* 0.8
${\dot{V}}_{CO_{2}}$ (mL min^−1^)	*F*_(1,117) _= 0.9, *P =* 0.3	*F*_(2,117) _= 0.2, *P =* 0.7	*F*_(1,117) _= 3.1, *P =* 0.07	***F*_(2,117) _= 3.1**, ***P =* 0.04**	*F*_(2,117) _= 0.02, *P =* 0.9	*F*_(1,117) _= 0.3, *P =* 0.5	*F*_(2,117) _= 2.9, *P =* 0.05
${\dot{V}}_{O_{2}}$ (mL min^−1^)	*F*_(1,117) _= 0.04, *P =* 0.8	*F*_(2,117) _= 0.05, *P =* 0.9	*F*_(1,117) _= 0.9, *P =* 0.3	*F*_(2,117) _= 1.3, *P =* 0.2	*F*_(2,117) _= 0.1, *P =* 0.9	*F*_(1,117) _= 0.003, *P =* 0.9	*F*_(2,117) _= 1.4, *P =* 0.2

*Note* that these measurements are not normalised to body mass (see text for details). Values in bold indicate a statistically significant result at *P <* 0.05.

Once the instruments were calibrated, the rat's weight and rectal temperature were measured and the animal was placed in the plethysmography chamber where it was allowed to acclimatise for ∼1 h. Experiments began when the rat was calm and the respiratory signal was stable. Respiratory activity was measured in room air (normoxia) for 3 h to allow sufficient time for reliable quantification of apnoeas during sleep (details below). Following this 3 h period, rats were exposed to hypoxic and hypercapnic challenges to assess the responsiveness of respiratory reflexes; in each experiment, the order of the stimulus was determined randomly. The hypercapnic response was tested by increasing CO_2_ levels in the inflowing gas mixture to 5%. Chamber CO_2_ level reached steady state within 2 min and stimulation was maintained for 10 min; the original recordings reported in Figure 3a illustrate the dynamics of the stimulation procedure and the ventilatory response. Ventilatory parameters were specifically analysed during the final 5 min of CO_2_ exposure to capture the steady state of the respiratory response (Fournier et al., 2015, 2014; Tenorio‐Lopes et al., 2017). The hypoxic challenge aimed to evaluate the peripheral (carotid body‐mediated) component of the hypoxic chemoreflex. Thus, hypoxia was induced by adding pure N_2_ to the inflowing gas so the O_2_ levels were rapidly reduced to 10% for 1 min; the original recordings reported in Figure 4a illustrate the dynamics of stimulation and ventilatory response. Note, the analysis focused on ventilatory responses during the hypoxic peak, defined as the 1‐min period when chamber O₂ concentration was maintained at ∼10% (Powell et al., 1998).

### 2.5 Quantification of apnoeas during non‐REM sleep

**FIGURE 1:**
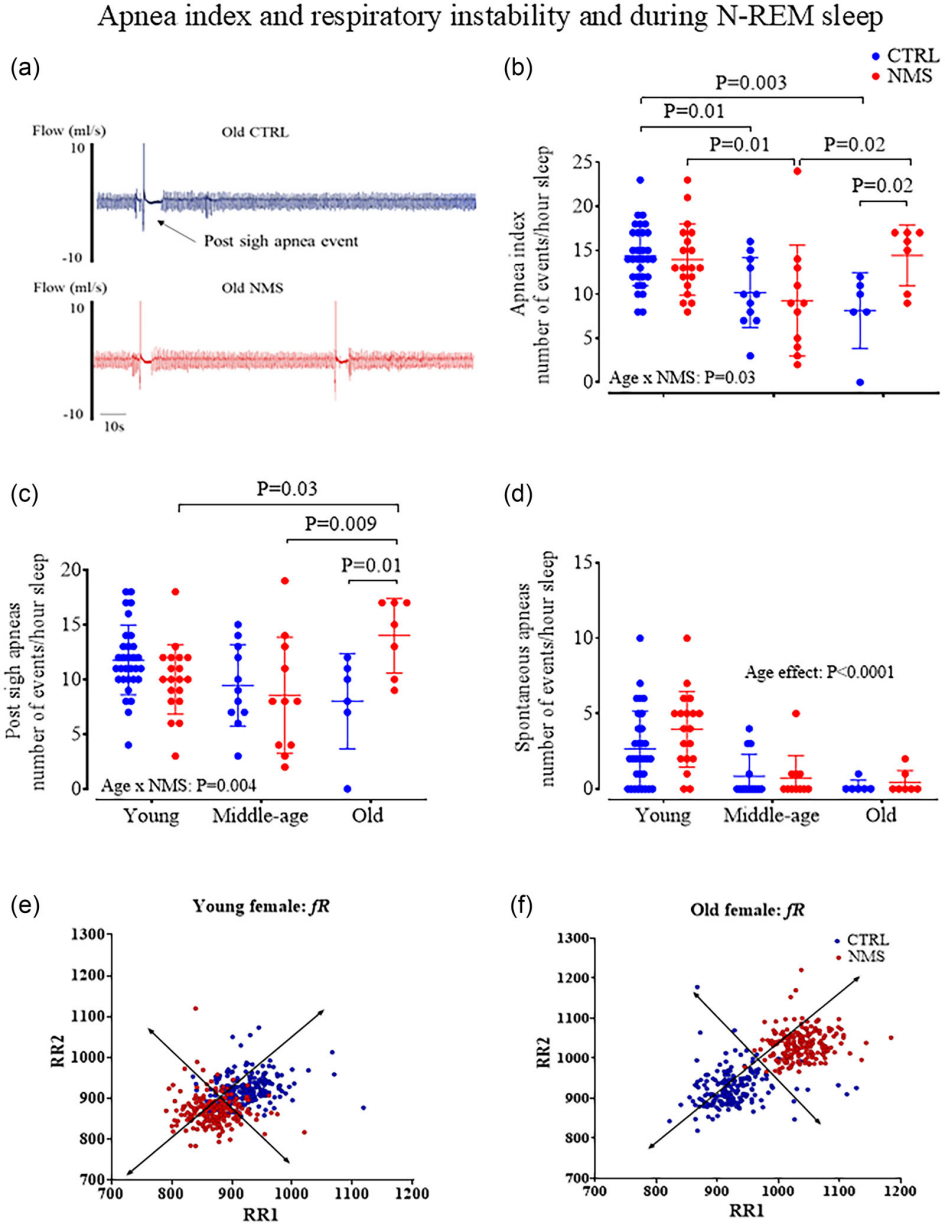
Age‐specific effect of NMS on the apnoea index during non‐REM sleep. (a) Original plethysmography recordings comparing respiratory activity during non‐REM sleep from old CTRL (blue) and NMS (red) female rats. (b) Population data comparing the apnoea index (total number of apnoeic events per hour of non‐REM sleep) across age groups and between CTRL and NMS females. (c) Frequency of post‐sigh apnoea. (d) Frequency of spontaneous apnoeas. Each animal is represented by an individual data point with mean ± SD. (e, f) Poincare plot analyses of respiratory viability in young (e) and old (f) females. Each individual point represents a breathing cycle in CTRL (blue) and NMS (red) rats. The ANOVA results are reported on panels (b–d).

During sleep, the loss of the wakefulness drive to breath reveals anomalies in respiratory control, including respiratory instability and apnoea. Changes in EEG and EMG signals are the gold standard to identify and discriminate sleep–wake states but because this approach requires chronic instrumentation, the stress associated with the surgical procedure was a concern, especially in old animals. However, identification of sleep–wake states using stability of the breathing signal offers a non‐invasive alternative that matches results obtained with EEG/EMG recording with 90% accuracy (Bastianini et al., 2017). Sleep–wake states were therefore identified using the criteria defined by (Bastianini et al., 2017) and recently validated by Ganouna‐Cohen et al. (2023). However, analysis of respiratory activity focused on non‐REM sleep; as shown in Figure 1a, this state is the most prevalent and easiest to identify as the regularity of breathing frequency (*f*_R_) and tidal volume are highest during this state.

Apnoeas were defined using the criteria commonly used in our group (Gagnon et al., 2023; Ganouna‐Cohen et al., 2023; Joseph et al., 2020). Briefly, apnoeas consist of respiratory pauses exceeding two typical respiratory cycles after a sigh (post‐sigh apnoeas) or without a preceding sigh (spontaneous apnoeas); sighs were identified by inspiratory volumes at least double the normal tidal volume (Figure 1a). Apnoeas were counted and expressed as the number of events per hour. The apnoea index consists of the total number of events (post‐sigh and spontaneous apnoeas) expressed per hour of non‐REM sleep. The duration of each apnoeic event was measured from the onset of the apnoea to the return of respiratory activity.

Breathing variability was examined using the Poincaré plot, a non‐linear geometric analysis measuring individual breath duration as variability indexes SD1 (short‐term) and SD2 (long‐term breathing variability) (Brennan et al., 2001). Variability indexes were assessed for three periods of ∼400 breaths during non‐REM sleep and were expressed as the mean.

### 2.6 Food intake, body morphology and composition as indicators of metabolism

Weekly assessments of food intake, body mass, and body length were conducted over one week to complement measurements of ${\dot{V}}_{O_{2}}$ and ${\dot{V}}_{CO_{2}}$ as indicators of metabolism. BMI was calculated as body weight (g)/length^2^ (cm^2^), with the length calculated from the nose‐to‐anus distance (Bernardis, 1970; Novelli et al., 2007). Food intake was measured by providing a known amount of food (500 g) to each cage. After 1 week, the remaining food was weighed, and the average amount consumed was divided equally between the two rats housed in the cage; results were then expressed as daily food intake (g/day) (Novelli et al., 2007). Because rats were housed in pairs, food consumption values represent an average intake per rat, assuming equal consumption between cage‐mates. While this approach introduces some variability, it provides a valid approximation for comparative purposes between groups.

Body composition was analysed using time‐domain nuclear magnetic resonance (TD‐NMR; Bruker Optics, Ettlingen, Germany). This instrument provides detailed measurements of fat, lean mass and fluids that compose the rat's body (Judge et al., 2008). The percentage of body fat, lean tissue and fluid was calculated by dividing their respective masses by the rat's body weight. All animals were compared at the same age across groups to ensure age‐matched assessments, allowing for accurate comparisons of metabolic indicators, body composition, and food intake trajectories across different life stages and experimental conditions.

### 2.7 Blood biochemistry: cytokine and leptin analyses

In rodents and humans, age‐related decline in neurological performance has been linked to altered plasma levels of cytokines, such as interleukin (IL)‐4, IL‐6, IL‐10, and tumour necrosis factor‐alpha (TNF‐α) (Scheinert et al., 2015). These cytokines help regulate immune responses and inflammation, which are closely linked to metabolic disorders and stress‐related conditions (Bhasin et al., 2022). Leptin is a peptide primarily synthesised and released by white adipose tissue to influence neural control of energy intake and metabolism (Ramos‐Lobo & Donato, 2017). Of note, leptin also influences respiratory control (Caballero‐Eraso et al., 2019). Importantly, both obesity and high leptin levels have been observed in patients with sleep apnoea (Patel et al., 2004).

Plasma cytokine levels were measured in 95 samples, distributed among three age groups: young, middle‐aged and old. For the young group, 60 samples were collected (39 from CTRL and 21 from the NMS group); this larger sample size allowed for the evaluation of the oestrous cycle effect. The middle‐aged group consisted of 17 samples (eight from CTRL and nine from NMS), while the old‐age group included 18 samples (10 from CTRL and 8 from NMS). Plasma leptin levels were analysed in 57 samples from all age groups: young (13 CTRL animals, 15 NMS), middle‐aged (8 CTRL, 9 NMS), and old (6 CTRL, 6 NMS).

Leptin and cytokine levels were measured using a multiplex bead chemiluminescence immunoassay, read using a Luminex200 (Bio‐Rad Laboratories Inc., Hercules, CA, USA). The measurements were conducted using the Bead Panel RECYTMAG‐65K kit (Bio‐Rad) (Kotzé‐Hörstmann et al., 2022; Scheinert et al., 2015). A minimum of 50 beads per analyte were measured using the Bio‐Plex Manager software (Bio‐Rad). All analyte kits were purchased from EMD Millipore (Billerica, MA, USA), and procedures were performed according to the manufacturer's protocols (Scheinert et al., 2015). Bio‐Plex Manager software was used to calculate calibration curves using a five‐parameter logistic curve fitting from recombinant standards. When cytokine levels were below the detection level of the assay, half of the lowest detectable values provided by the manufacturer were added to the dataset for statistical purposes. According to the ROUT outlier identification test with *Q* = 1%, outliers were excluded, representing 0 values for IL‐4, eight values for IL‐1β, three values for IL‐6 and nine values for IL‐10. TNF‐α was not detectable across all groups and ages, so it was excluded from the analysis. Undetectable cytokine levels in young and middle‐aged rats were attributed to concentrations falling below the assay detection limit, rather than technical problems in sample processing. Cytokine data were transformed using a logarithmic scale to visualise distributions stratified by age (Koelman et al., 2019). All statistical analyses were performed on the raw (non‐log‐transformed) data. The log transformation was applied for visualization purposes in the figure to better illustrate the distribution of cytokine levels while maintaining a reasonable scale.

### 2.8 Immunohistochemical analysis of the caudal nucleus of the solitary tract

As it receives numerous sensory afferents relevant to cardiorespiratory homeostasis, the caudal nucleus of the solitary tract (cNTS) plays a key role in reflexive respiratory adjustments to ensure homeostasis of arterial blood gases (Figure 7a). Activation of leptin receptors present in this structure contributes to the stimulatory actions of leptin on respiratory function (Amorim et al., 2022; Yu et al., 2021). Consequently, the cNTS has been linked to the development of cardiorespiratory and metabolic dysfunctions observed in SA and metabolic disorders (Zoccal et al., 2014). Here, we compared basal level of neuronal activation (FosB) and the number of neurons present in the structure (NeuN). Coronal sections (40 µm) of frozen brains were prepared using a cryostat, and sections containing the cNTS were processed for immunohistochemistry. Unfortunately, due to technical problems, brains from the middle aged group could not be used in those experiments.

#### 2.8.1 FosB immunolabeling

FosB is a transcription factor commonly used as a marker of chronic neuronal activation. Immunolabeling was therefore conducted in the cNTS sections using an established free‐floating protocol (Ambrozio‐Marques et al., 2023; Ansorg et al., 2015). Briefly, animals were deeply anaesthetized with isoflurane and perfused intracardially with phosphate‐buffered saline (PBS; 1 mL g^−1^ body weight, pH 7.4), followed by 4% paraformaldehyde (PFA) in PBS at the same volume ratio (1 mL g^−1^ body weight). After perfusion, brains were carefully extracted, post‐fixed in 4% PFA at 4°C for 48 h, and then transferred to a 30% sucrose solution for cryoprotection, also at 4°C for 48 h. Following this process, brains were frozen on dry ice and stored at −80°C until sectioning.

Frozen brains were cut into 40 µm coronal slices using a sliding microtome with the ‘dry ice’ method (Anders, 1942). These sections were then placed in a cold cryoprotectant solution consisting of 0.05 M sodium phosphate buffer, 30% ethylene glycol and 20% glycerol, and stored at −20°C. Free‐floating tissue sections were initially rinsed in Tris‐buffered saline (TBS), pH 7.4, for 1 min at room temperature. Throughout the procedure, tissue plates were kept on an orbital shaker (Vevor Adjustable Variable Speed Oscillator Orbital Rotator Shaker, Industry, CA, USA) at 20 rpm. Antigen retrieval was achieved by incubating sections in a solution of 0.1 M citric acid and 0.1 M sodium citrate (pH 6.0), first at 65°C for 5 min, then at 95°C for 10 min (Yamashita & Katsumata, 2017). Sections were allowed to cool to room temperature for 30 min, followed by three 5‐min washes in TBS. Endogenous peroxidase activity was blocked with a 3% hydrogen peroxide solution in TBS for 30 min, followed by another three 5‐min TBS washes. To reduce non‐specific binding, sections were incubated for 1 h in a blocking solution containing 1% BSA and 0.4% Triton X‐100 in TBS. The sections were then incubated overnight at 4°C with the primary antibody for FosB (cat. No. 2251, Cell Signaling Technology, Danvers, MA, USA) at a dilution of 1:2000 in the blocking solution.

On the following day, the sections were brought to room temperature for 1 h, washed three times in TBS (5 min each), and then incubated with a biotinylated secondary antibody (goat anti‐rabbit IgG, 1:400 dilution in blocking solution, Vector Laboratories, Burlingame, CA, USA) for 3 h at room temperature. Next, sections were treated with the Vectastain Elite avidin‐biotin complex (ABC) for 90 min (Ansorg et al., 2015). After another series of TBS washes (three 5‐min washes), the sections were developed using the nickel chloride‐enhanced diaminobenzidine (DAB) peroxidase reaction (SigmaFast DAB with metal enhancer, Sigma‐Aldrich, St Louis, MO, USA) for 4 min to reveal the biotinylated antibody. After a final series of TBS washes (three 5‐min washes), the sections were mounted onto Fisherbrand Tissue Path Superfrost Plus Gold slides (Thermo Fisher Scientific, Waltham, MA, USA), dried for 48 h, and coverslipped with a permanent mounting medium, preparing them for microscopic examination. Slides with the FosB immunolabelled tissue were visualized under a microscope (Eclipse E600, Nikon, Tokyo, Japan) equipped with a camera (Infinity 3, Lumenera Corporation, Ottawa, ON, Canada) and stored as digital images. Images used for data analysis were captured with a ×10 magnification objective. Images were digitally acquired into ImageJ (NIH, Bethesda, MD, USA; RRID: SCR_003070). All images were filtered and adjusted equally for contrast and light levels for clarity. Analyses were performed in the caudal region of the NTS (bregma: −13.68 to 14.30 mm) based on illustrations from the rat brain stereotaxic atlas (Paxinos & Watson, 1998). The analyses were conducted in a randomized order, and the experimenters were blinded to the identities of the treatments. For each section, the right and left sides were quantified and the results averaged. The number of immunopositive cells was expressed as a function of the size of the structure (cell density) using standardized templates that were built according to the specific form of the structure. Figure 7b shows a schematic representation of the templates and representative photomicrographs.

#### 2.8.2 NeuN staining

NeuN is a specific marker of post‐mitotic neurons. NeuN immunolabeling was performed on cNTS PFA‐fixed free‐floating sections that were first washed in PBS; non‐specific antibody binding was blocked with 10% normal donkey serum (NDS, Millipore) in 0.3% Triton–PBS. All sections were then incubated overnight at room temperature with primary antibody for NeuN (mouse, 1:500, Millipore cat. No. MAB377) (Humphrey et al., 2023). Sections were then washed and incubated for 2 h with secondary antibodies for Alexa Fluor, Alexa Fluor 594 (donkey anti‐mouse, 1:200, Jackson ImmunoResearch Laboratories, West Grove, PA, USA, cat. no. 715‐586‐151). Tissues were mounted, air‐dried, and coverslipped using ProLong Diamond (P36962, Thermo Fisher Scientific). Images of the sections were taken with a digital camera (optiMOS, QImaging) on a conventional fluorescence microscope (BX51WI, Olympus) with an attached LED lamp (pE‐300white, CoolLED) for illumination. Exposure times remained constant within same‐age groups to allow comparison of fluorescent intensity. Images were uniformly postprocessed and analysed using Fiji software (ImageJ 1.53t, NIH) (Humphrey et al., 2023). Neurons were manually counted by two investigators blinded to the experimental condition in a 150 × 150 µm box within the region of interest. Staining intensity was normalized to the control in the same age group.

### 2.9 Statistics

As mentioned previously, the fact that body mass does not scale linearly across groups impacts analysis of basal (normoxic) respiratory variables (${\dot{V}}_{E}$, *V*_T_, *f*_R_, ${\dot{V}}_{O_{2}}$ and ${\dot{V}}_{CO_{2}}$), which are usually normalised to body weight. To address this issue, the effect of body mass was considered by analysis of covariance. For all other variables, detailed statistical analyses were conducted using two‐way ANOVA to analyse the interactions and main effects of treatments across different age groups in CTRL and NMS animals. Following ANOVA, *post hoc* comparisons were made using the Holm–Šidák multiple comparisons test. Statistical significance was predetermined at *P* ≤ 0.05. All statistical analyses were conducted using Prism (Version 8.4.2, GraphPad Software, San Diego, CA, USA). Note that to facilitate reading and improve clarity, the results of ANOVA and *post hoc* tests are reported in the figures.

## 3 RESULTS

### 3.1 Age‐specific effect of NMS on the apnoea index during non‐REM sleep

In control females, the apnoea index progressively decreased with age; however, NMS altered this trajectory as the apnoea index of old NMS females was 75% greater than that of age‐matched CTRL (Figure 1a, b). As the majority of apnoeas occurred after a sigh, the frequency of those events had the highest impact on the apnoea index (Figure 1c). The frequency of sighs (with and without apnoeas) showed a significant age × NMS interaction (*P =* 0.03), but overall sigh frequency remained comparable between CTRL and NMS groups within each age group (data not shown). Spontaneous apnoeas were less frequent and ageing reduced their occurrence by 50%, but they were not influenced by NMS (Figure 1d). The duration of apnoeas was similar between groups (data not shown). Poincaré plots were performed to compare stability of respiratory signals between groups (Figure 1e, f). Quantification of the short‐term (SD1) and long‐term (SD2) variability did not reveal significant differences between CTRL and NMS rats at either age (data not shown).

### 3.2 Respiratory measurements

#### 3.2.1 Normoxia

**FIGURE 2:**
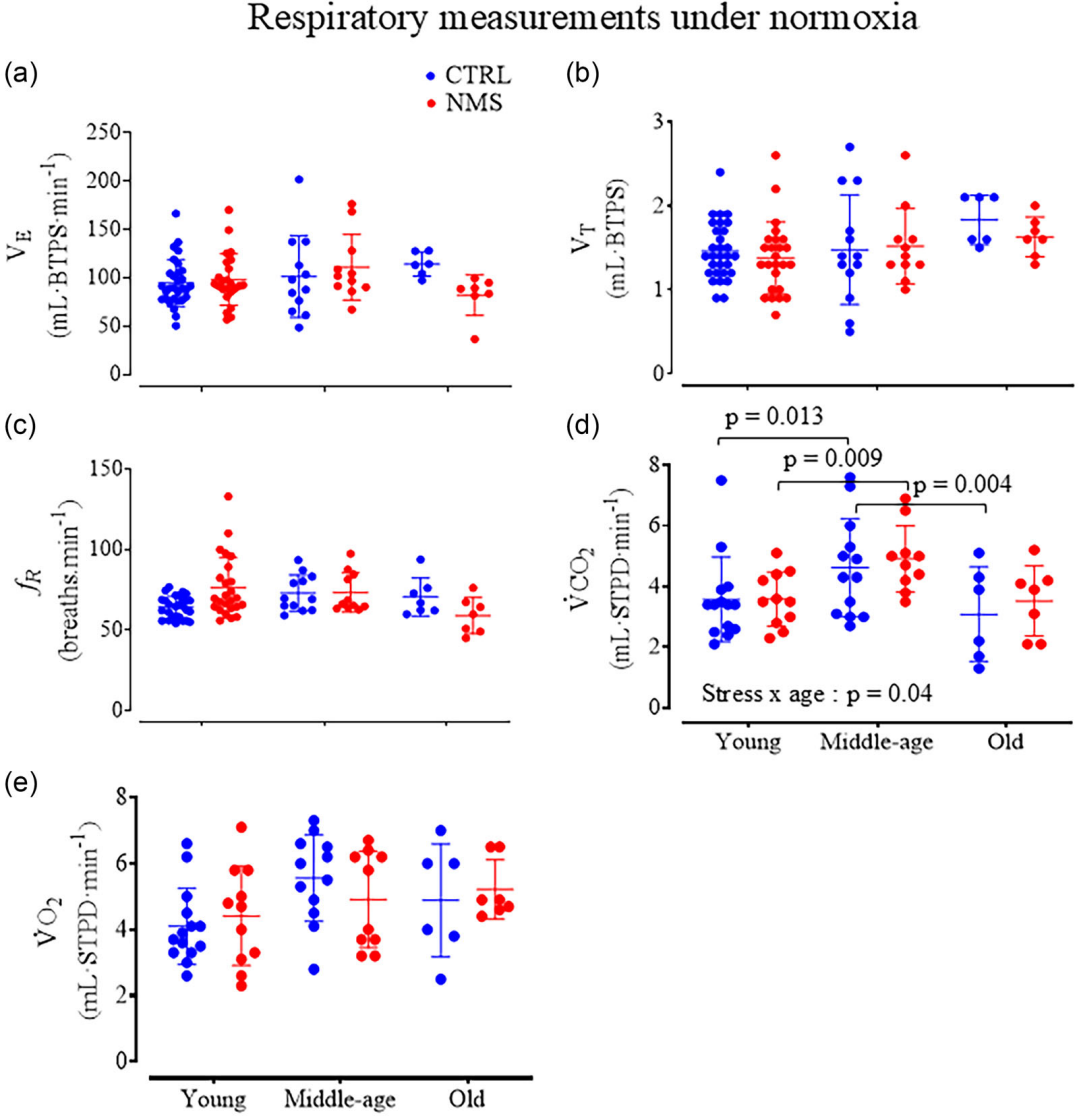
Respiratory measurements under resting (normoxic) conditions. Individual data points and group means ± SD are shown for (a) minute ventilation (${\dot{V}}_{E}$, mL BTPS min⁻¹); (b) tidal volume (*V*_T_, mL BTPS); (c) breathing frequency (*f*_R_, breaths min⁻¹), (d) Carbon dioxide production (${\dot{V}}_{CO_{2}}$, mL STPD min⁻¹), and (e) O_2_ consumption (${\dot{V}}_{O_{2}}$, mL STPD min⁻¹) for each age group. Note that these data are not normalized to body mass (see text for details); however, tidal volume and ${\dot{V}}_{E}$ were normalized to body temperature, atmospheric pressure, saturated (BTPS). CO_2_ production was calculated and normalized to standard temperature pressure dry (STPD). The CTRL animals are in blue and the NMS in red. ANOVA results arereported in Panel D.

Resting values for ${\dot{V}}_{E}$, *V*_T_, *f*_R_, ${\dot{V}}_{O_{2}}$ and ${\dot{V}}_{CO_{2}}$ for both CTRL and NMS females across age groups are reported in Figure 2; note that these values are not normalised to the animal's weight. Considering body mass, along with stress and age as fixed factors in an ANCOVA revealed no effect of NMS or age for most variables (Table 1). For ${\dot{V}}_{CO_{2}}$, however, the values measured in old females were lower than at middle age. This drop was significant in CTRL, but not NMS, thus indicating that the influence of NMS on metabolic rate differs with age. The ventilatory equivalents ${\dot{V}}_{E}$/${\dot{V}}_{O_{2}}$ and ${\dot{V}}_{E}$/${\dot{V}}_{CO_{2}}$ declined with age in NMS females but not CTRL (Table 2). These observations support that the age‐related changes in respiratory function observed in NMS rats are independent of body mass.

**TABLE 2:** Metabolic parameters measured in female CTRL and NMS rats across different ages under normoxic conditions.

	Control (CTRL)			Neonatal maternal separation (NMS)					
	Young	Middle‐age	Old	Young	Middle‐age	Old	NMS effect	Age effect	Interaction
${\dot{V}}_{E}$/${\dot{V}}_{O_{2}}$	22 ± 4	20 ± 9	26 ± 10	23 ± 5	20 ± 7	15 ± 4^a,b^	*P =* 0.06, *F*_(1,55) _= 3.7	*P =* 0.2, *F*_(2,55) _= 1.5	***P =* 0.02**, ***F*_(2,55) _= 4.3**
${\dot{V}}_{E}$/${\dot{V}}_{CO_{2}}$	24 ± 6	24 ± 7	37 ± 18	30 ± 6	22 ± 7	24 ± 8^a^	*P =* 0.2, *F*_(1,55) _= 1.7	*P =* 0.03, *F*_(2,55) _= 3.6	***P =* 0.01**, ***F*_(2,55) _= 4.7**
${\dot{V}}_{CO_{2}}$/${\dot{V}}_{O_{2}}$	0.9 ± 0.2	0.9 ± 0.2	0.6 ± 0.1	0.8 ± 0.1	0.8 ± 0.2	0.6 ± 0.1	*P =* 0.3, *F*_(1,55) _= 1.0	***P =* 0.0006**, ***F*_(2,55) _= 8.8**	*P =* 0.2, *F*_(2,55) _= 0.2
Body temperature (°C)	37.2 ± 0.4	37.8 ± 0.3	37.5 ± 0.4	37.2 ± 0.7	37.9 ± 0.3	37.5 ± 0.4	*P =* 0.8, *F*_(1,55) _= 0.1	***P <* 0.0001**, ***F*_(2,55) _= 12.5**	*P =* 0.9, *F*_(2,55) _= 0.1
Body mass (g)	263 ± 35	320 ± 45^b^	381 ± 80^b,c^	305 ± 43	381 ± 37^b^	436 ± 62^b,c^	***P <* 0.0001**, ***F*_(1,65) _= 18.3**	***P <* 0.0001**, ***F*_(2,65) _= 39.9**	*P =* 0.7, *F*_(2,65) _= 0.7

Data are presented as means ± SD. Statistical analyses were performed on raw data. Comparisons were conducted using two‐way ANOVA, followed by the Holm‐Šidák *post hoc* test when applicable. Significant differences are indicated as follows: ^a^significantly different from CTRL at the same age; ^b^significantly different from young within the same treatment group; ^c^significantly different from middle‐aged within the same treatment group.

#### 3.2.2 Hypercapnic ventilatory response

**FIGURE 3:**
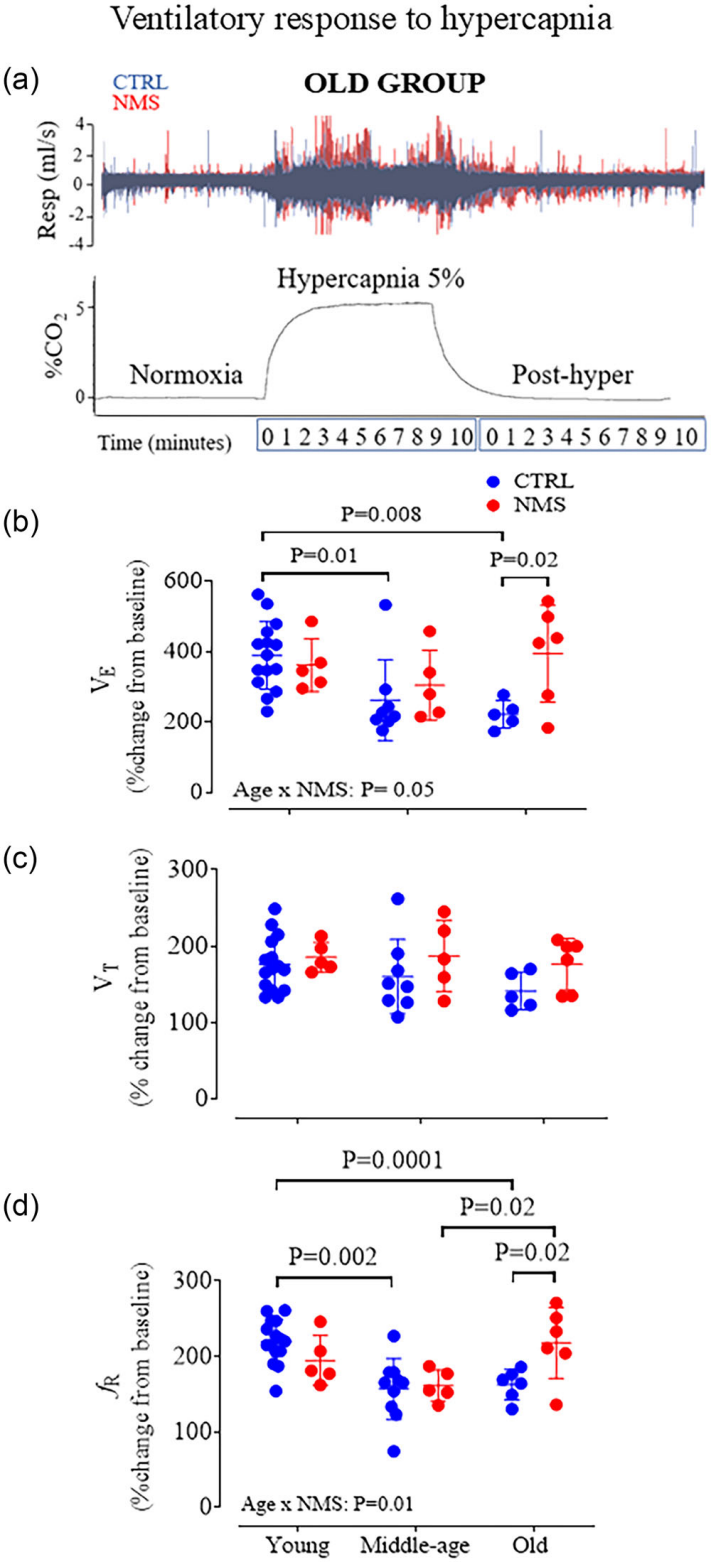
Hypercapnic ventilatory response. (a) Top: original plethysmography recordings comparing the hypercapnic ventilatory responses of old rats that were either maintained in control conditions (CTRL; blue) or subjected to neonatal maternal separation (NMS; red). Bottom: CO_2_ level in the plethysmography chamber during the protocol. (b) ${\dot{V}}_{E}$ , (c) *v*_t_ , and (d) *f*_r_ responses expressed as percentage change from baseline. Each individual point represents a value from a CTRL (blue) or NMS rat (red). Each animal is represented by an individual data point with mean ± SD. The ANOVA result is reported on panels (b and d).

Exposure to hypercapnia stimulated ${\dot{V}}_{E}$ in all rats owing to significant increases in *f*_R_ and *V*_T_ (Figure 3a). Control females experienced an age‐dependent decline in the intensity of their response to CO_2_, whereas the NMS group maintained a relatively high ${\dot{V}}_{E}$ response. At old age, the response measured in NMS is 172% higher than in CTRL (Figure 3b). As the *V*_T_ response was unaffected by age or NMS (Figure 3c), the age‐dependent effect of NMS on the hypercapnic ventilatory response was mainly mediated by a greater increase in *f*_R_ (Figure 3d).

#### 3.2.3 Hypoxic ventilatory response

**FIGURE 4:**
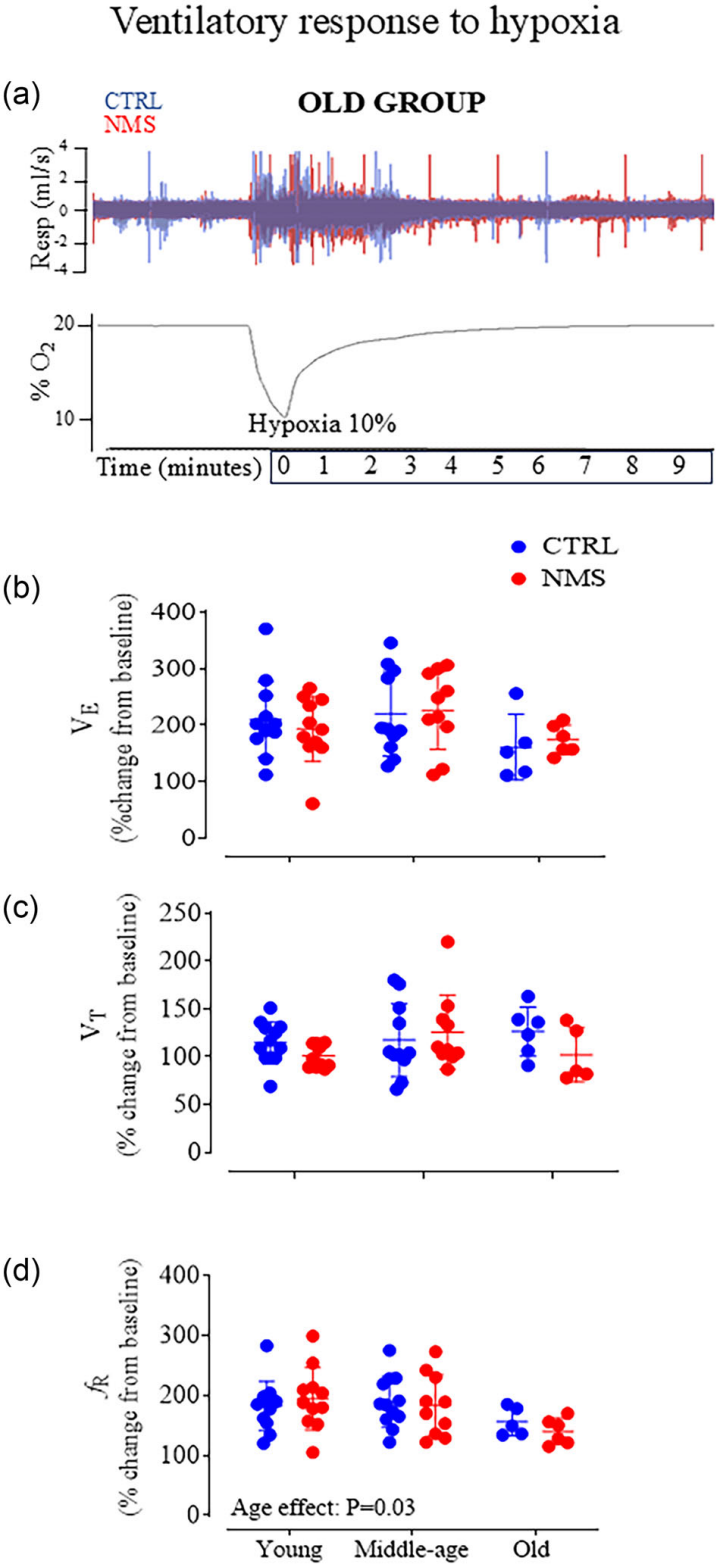
Hypoxic ventilatory response. (a) Top: original plethysmography recordings comparing the hypoxic ventilatory responses of old rats that were either maintained in control conditions (CTRL; blue) or subjected to neonatal maternal separation (NMS; red). Bottom: O_2_ level in the plethysmography chamber during the protocol. (b) ${\dot{V}}_{E}$ , (c) *V*_T_ , and (d) *f*_R_ responses expressed as percentage change from baseline. Each individual point represents a value from a CTRL (blue) or NMS rat (red). Each animal is represented by an individual data point with mean ± SD. The ANOVA result is reported on panels d.

Acute exposure to hypoxia stimulated breathing in all animals (Figure 4a). The intensity of the ${\dot{V}}_{E}$ response was not altered by NMS or age (Figure 4b). The *V*_T_ response was unaffected by either factor; while the frequency response decreased slightly with age, this was not sufficient to affect ${\dot{V}}_{E}$ significantly (Figure 4b, c, d, respectively).

### 3.3 Morphology, body composition, and food consumption

**FIGURE 5:**
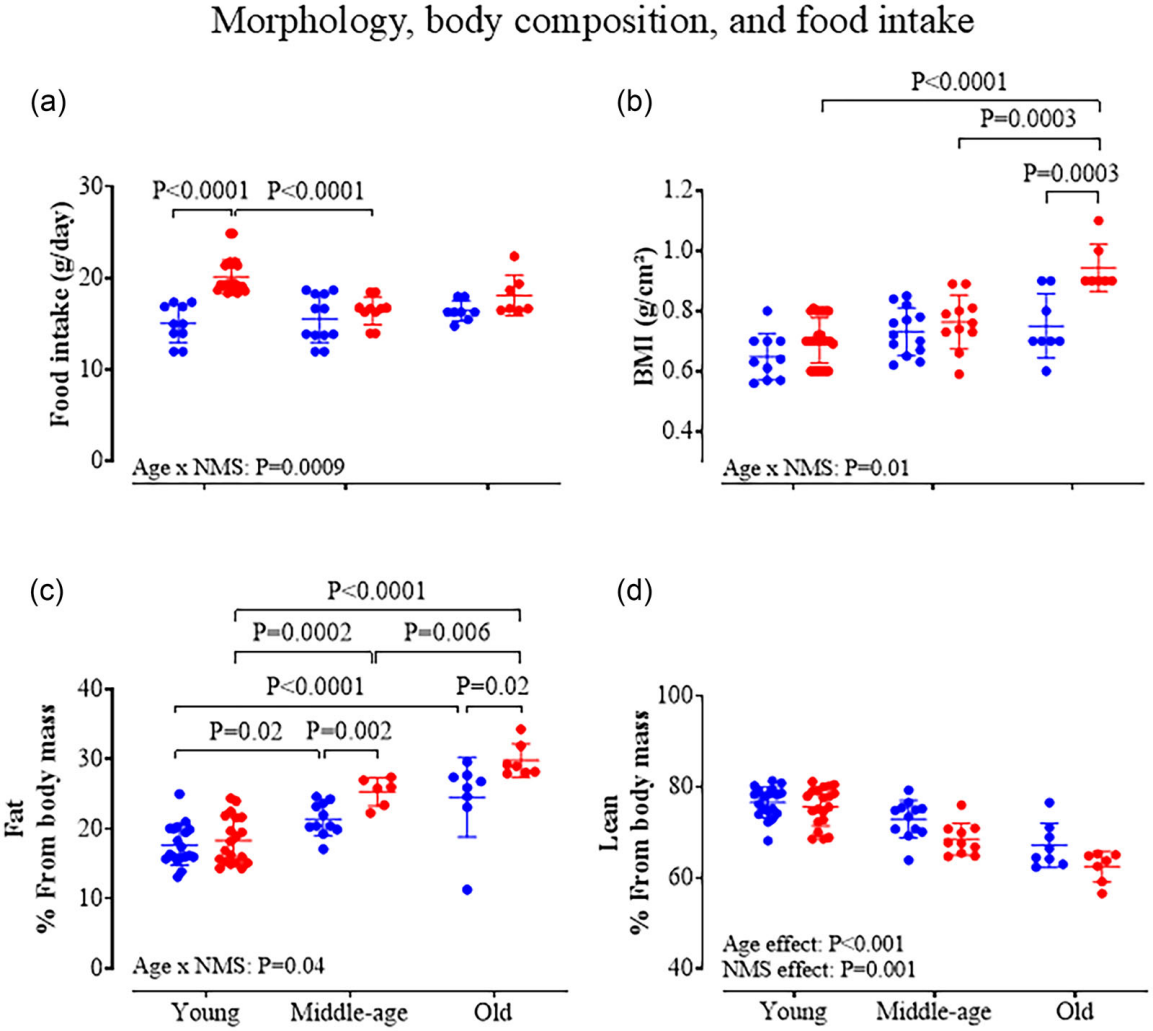
Morphology, body composition, and food intake. Selected indicators of metabolism were measured including: (a) Food intake, calculated over a 1‐week period and expressed as daily food intake (g/day). (b) Body mass index, calculated as the ratio of body size over weight. Nuclear magnetic resonance was used to measure (c) percentage of fat and (d) lean mass. Each individual point represents a value from a CTRL (blue) or NMS rat (red). Each animal is represented by an individual data point with mean ± SD. The ANOVA result is reported on the panels.

The food intake of CTRL animals was relatively stable across age groups; in young NMS females, however, food consumption was 34% greater than CTRL; however, this difference did not persist into middle or old ages (Figure 5a). Body mass (Table 2), size, body mass index (BMI) and fat composition increased with age; however, NMS exacerbated the age‐related differences (Table 2, Figure 5). Specifically, NMS females weighed between 15% and 19% more that age‐matched CTRLs (Table 2). These changes affected BMI and the most important difference was observed in old age where the BMI of NMS rats was 24% greater than that of the CTRL group (Figure 5c). Consistent with those changes, the proportion of lean mass decreased with age, especially in NMS females (Figure 5d).

### 3.4 Effects of age and NMS on circulating leptin and cytokines

**FIGURE 6:**
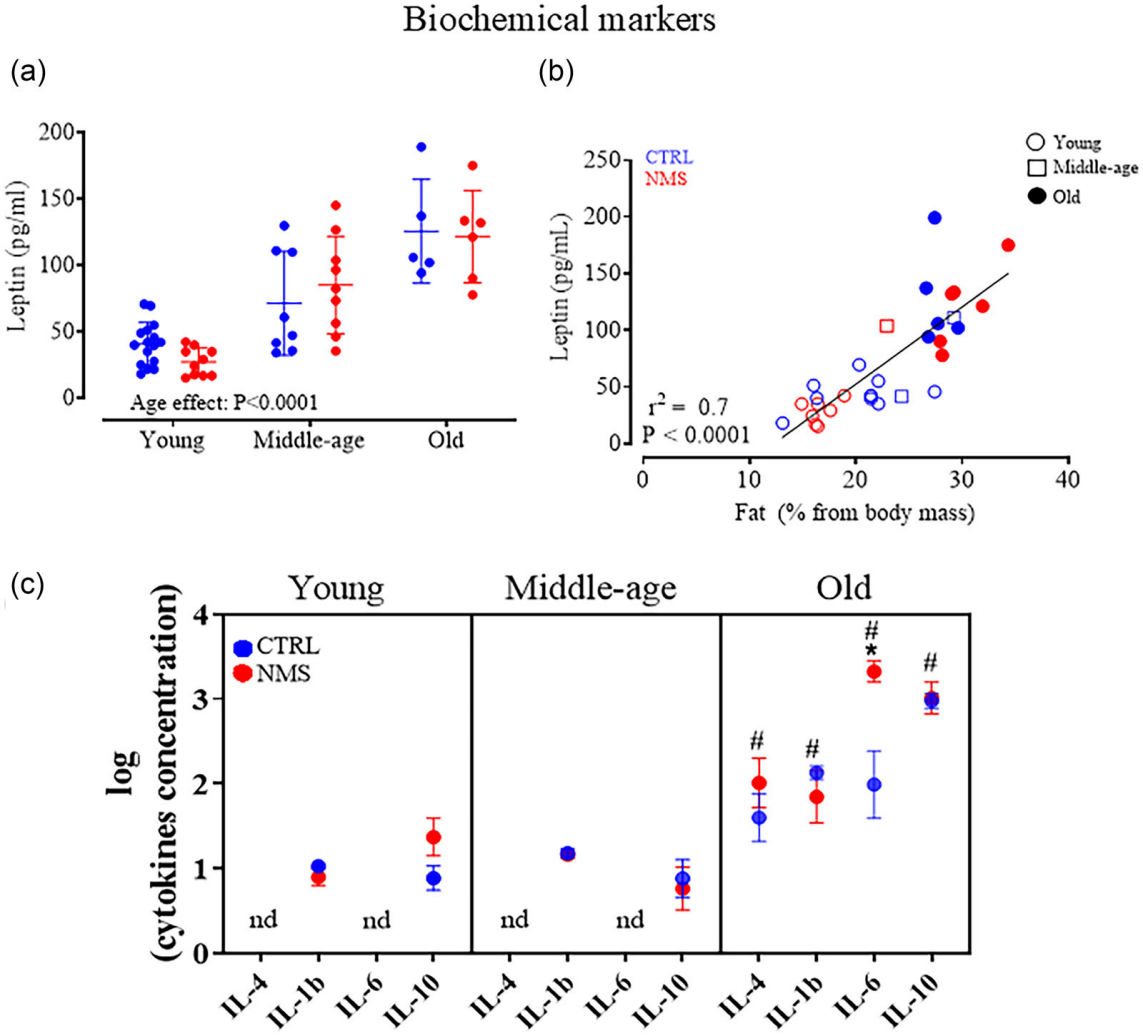
Effects of age and NMS on circulating leptin and cytokines. (a) Comparison of plasma leptin levels across age groups and between CTRL and NMS females. (b) Correlation between relative body fat mass and circulating leptin in young (open circles), middle‐age (open squares) and old females (filled circles). Data are reported for CTRL (blue) and NMS (red). (c) Comparison of plasma cytokine levels (IL‐4, IL‐1β, IL‐6, IL‐10) between CTRL and NMS animals at young, middle‐age and older groups. Values are expressed on a logarithmic scale. ANOVA was performed on raw (non‐logarithmic data); # indicates a value significantly different from corresponding young value at *P* ≤ 0.05 in the graph. When cytokine levels were below the detection level of the assay (nd, not detectable), half of the lowest detectable values provided by the manufacturer were added to the dataset for statistical purposes. ANOVA result is reported in panel a. *Value significantly different from corresponding control value at *P* ≤ 0.05.

Produced by adipocytes, leptin plays a major role in energy balance and stimulates breathing (Pham et al., 2022). Plasma leptin levels increased progressively with age (Figure 6a) and the values were positively correlated with the percentage of fat that composes the rat's body (Figure 6b). These observations were not influenced by NMS.

By comparison with older females, cytokine levels were lower in young and middle‐aged groups (Figure 6c). The concentration of IL‐6 in old animals was 765 ± 1347 pg mL^−1^ for CTRL animals and notably higher at NMS 2427 ± 1038 pg mL^−1^. A significant NMS effect was observed for IL‐6 (*t*‐test: *P =* 0.02). For IL‐10, the concentrations were 1251 ± 721 pg mL^−1^ in CTRL and 1552 ± 1166 pg mL^−1^ in NMS animals; these values were unaffected by NMS. These results highlight that ageing significantly impacts cytokine levels, particularly IL‐1β, IL‐6 and IL‐10, in old animals, with NMS treatment specifically elevating IL‐6 levels in this age group.

### 3.5 Immunohistochemistry of cNTS

**FIGURE 7:**
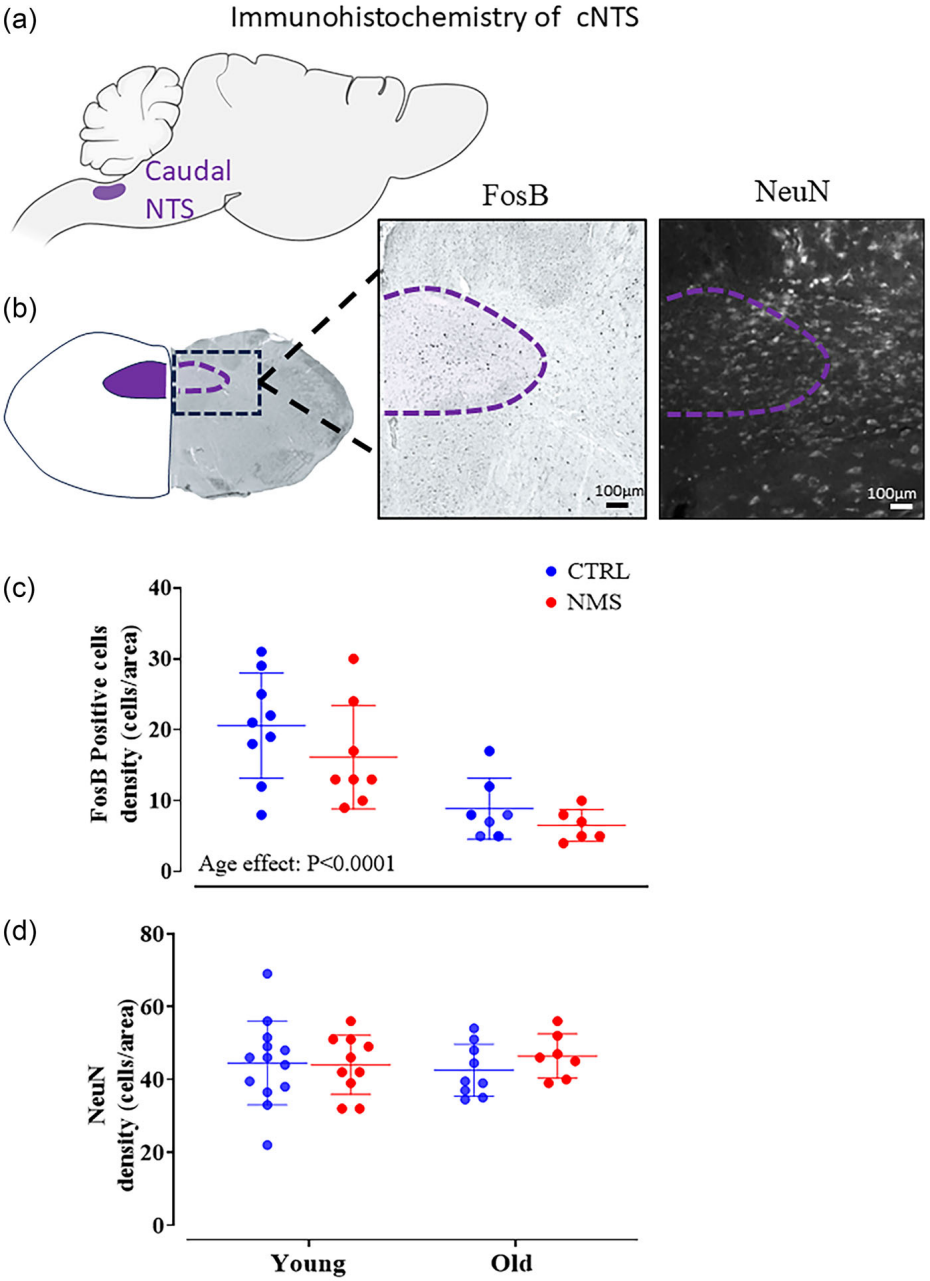
Immunohistochemistry of cNTS. (a) Sagittal view of the rat brain, illustrating the location of the cNTS. (b) Left panel has the coronal view illustrating the location of the cNTS. The dashed line indicates the location of the tissue sections. Middle panel has a representative photomicrograph stained for FosB, showing the region of interest. Right panel has a representative photomicrograph stained for NeuN, showing the region of interest. (c) Density of FosB‐positive cells. (d) Density of NeuN‐positive cells. Each individual point represents a value from a CTRL (blue) or NMS rat (red). Each animal is represented by an individual data point with mean ± SD. The ANOVA result is reported on panels c.

Altered basal activity of respiratory centres, like the cNTS, is indicative of increased afferent inputs converging onto this structure. The number of FosB expressing perikaryas in the cNTS significantly decreased with age but was not affected by NMS (Figure 7d). Conversely, the number of NeuN‐positive cells remained constant in all groups (Figure 7e), thereby indicating that the result observed for FosB was not due to a drop in the neuron population in this structure.

## 4 DISCUSSION

For ageing women, sleep apnoea (SA) is an important health issue owing to its broad impacts on physical and mental health; unfortunately, SA remains underestimated and understudied, especially in women (Kinkead et al., 2021). Although physiological changes during ageing are common, the manifestation of pathological conditions such as SA does not occur uniformly across all individuals. Elucidating the factors compromising the ageing trajectory in respiratory health is a fundamental physiological question with clear clinical implications. To address this important gap in knowledge (and its sex‐based inequity), we proposed that in females, ageing reveals the latent deleterious effects of early life stress on respiratory control. Our data strongly support this hypothesis as we demonstrate that old females subjected to neonatal maternal separation (NMS) develop a physiological phenotype in line with that of SA patients, namely, increased apnoeas during sleep with excessive chemoreflex responses and metabolic dysfunction. NMS females also exhibited elevated circulating IL‐6 levels under basal conditions, indicating the presence of persistent low‐grade inflammation even in the absence of a specific immune response to an external trigger. This chronic inflammatory state may further exacerbate metabolic and respiratory dysfunctions associated with ageing, contributing to the complex pathophysiology observed in late‐onset sleep apnoea.

### 4.1 Age and NMS‐related respiratory control disturbances

The progressive age‐related drop in apnoea index observed in CTRL females parallels the tendency for an age‐related reduction in breathing frequency; however, the elevated apnoea index observed in NMS females points to anomalies in respiratory control, as were observed during normoxia and acute CO_2_ challenge. The lack of significant differences in SD1 and SD2 confirms that the observed changes in apnoea index and breathing patterns are not attributable to altered breath‐by‐breath variability. By comparison with old CTRL females, aged NMS rats hypoventilated with respect their metabolic demand, as indicated by the lower ventilatory equivalents for both oxygen (${\dot{V}}_{E}$/${\dot{V}}_{O_{2}}$) and carbon dioxide (${\dot{V}}_{E}$/${\dot{V}}_{CO_{2}}$); during hypercapnic challenge, however, these females were more responsive than controls. These effects of NMS can contribute to the higher apnoea index reported in older NMS females (Böing & Randerath, 2015). Because O_2_ saturation was not measured in our animals, we do not know if those apnoeic events were physiologically significant, yet NMS induced other physiological disturbances.

Together with the hypoventilation, the increase in BMI and fat composition observed in old NMS females is in line with the phenotype reported in patients with obesity hypoventilation syndrome (OBS), a population in which 70% also suffer from obstructive apnoea (Amorim et al., 2022). Detecting airway obstructions with certainty requires concomitant monitoring of inspiratory muscles. Existing evidence in rodent models suggests that post‐sigh apnoeas are typically central, rather than obstructive, in nature (Davis & O'Donnell, 2013).

Because obstructive events are rare in rodents, we opted for a non‐invasive (and less stressful) approach to measure breathing such that it was not possible to determine if rats generated inspiratory efforts during apnoeas. While the parallels between old NMS females with OBS are of great interest, an enhanced CO_2_ response is contrary to what is reported in that population. That said, leptin resistance contributes to obesity and in obese (*ob*/*ob*) mice, leptin supplementation alleviates a reduced hypercapnic ventilatory response (Amorim et al., 2022; Berger et al., 2019). As adipocytes are the primary source of leptin, the values reported here are consistent with overall age‐related increase in body fat, yet the correlation analysis (Figure 6b) and the elevated CO_2_ response observed in NMS females do not support a functional change in leptin signalling. Exposure to an additional stress (e.g. high fat diet) may be necessary to develop this aspect of the phenotype.

### 4.2 NMS increases the risk of obesity and disrupts metabolic health

Early life stress has persistent and sex‐specific effects on the development of the hypothalamo–pituitary–adrenal (HPA) axis that contribute to the elevated risk of disease at various life stages (Shonkoff et al., 2009). The HPA axis is mainly known for its ability to orchestrate the stress response, but considering that it also regulates energy balance (Ulrich‐Lai & Ryan, 2014), it is not surprising that in human populations, early‐life adversity has been associated with increased risks for obesity and metabolic syndrome (Kivimäki et al., 2023; Scott et al., 2012; Tamashiro et al., 2011, 2005). The age‐dependent rise in body weight, body fat and BMI reported in NMS females is in line with clinical observations and indicates that, in addition to respiratory disturbance, NMS increases the risk of developing obesity and metabolic dysfunction, which are the main comorbidities of SA. An important factor that distinguishes obesity from respiratory outcomes is the direct contribution of behaviour to the problem. Owing to its impacts on neural control of metabolism and the reward system, chronic stress stimulates food intake, which, along with its impacts at the cellular level, will ultimately favour weight gain and fat accumulation (Adam & Epel, 2007; Torres & Nowson, 2007). Elevated food consumption was only observed in young NMS females, but the changes observed in older animals suggest that the transient change in behaviour (and/or its metabolic impacts) had a detrimental effect on BMI and body composition that persisted in time. These effects may contribute to higher apnoea index reported in old NMS females owing to the association between obesity, metabolic dysregulation and sleep apnoea (Peppard et al., 2000; Schwartz et al., 2008). Obesity mainly favours obstructive apnoeas, especially in men, but as mentioned previously, we do not know if apnoeic events were obstructive.

### 4.3 Hormonal and inflammatory biomarkers

Ageing is naturally associated with increased levels of inflammatory cytokines in circulation (Álvarez‐Rodríguez et al., 2012; Bruunsgaard et al., 2001; Krabbe et al., 2004). This age‐related increase in inflammatory activity, termed ‘inflamm‐aging’, is characterized by 2‐ to 4‐fold elevations in cytokines like TNF‐α, IL‐6 and IL‐1β (Krabbe et al., 2004). Multiple factors contribute to this low‐grade inflammation, including increased adipose tissue, decreased sex steroid production and subclinical infections. The results reported here are consistent with this notion, and while sex steroids were not measured in this study, previous work in our laboratory showed that sex steroids decrease in ageing females, but were not affected by NMS (Fournier et al., 2015). Thus, the greater proportion of body fat may explain the higher levels of IL‐6 observed in old NMS females. Increased levels of other inflammatory cytokines, such as TNF‐α, are often reported in a pro‐inflammatory state. However, it is unclear why we were unable to detect TNF‐α levels in our study even though cytokines were measured the same way. Despite this limitation, the preliminary assessment of the cytokine profile suggests that NMS favoured the emergence of a pro‐inflammatory status in old age. This inflammation, in turn, can interfere with several processes and exacerbate conditions such as metabolic dysfunction (Hotamisligil, 2006; Lumeng & Saltiel, 2011) and may also affect other physiological systems, potentially compounding age‐related decline in health (Chung et al., 2011; Franceschi & Campisi, 2014; Kiecolt‐Glaser et al., 2003; Puzianowska‐Kuźnicka et al., 2016). Elevated IL‐6 is a matter of concern as it is associated with many diseases of old ages, such as cardiovascular disease, osteoporosis, type 2 diabetes, obesity and certain cancers (Chung et al., 2011; Franceschi & Campisi, 2014; Kiecolt‐Glaser et al., 2003; Puzianowska‐Kuźnicka et al., 2016).

### 4.4 Limitations and conclusions

The fact that allometric corrections do not apply uniformly between groups is an interesting but unexpected observation. Without this normalisation, however, direct comparison of our results with established knowledge regarding the impact of ageing on respiratory function is challenging. While this raises important questions regarding the impact of environmental factors on the relationship between body mass and respiratory variables, addressing this issue adequately is clearly beyond the scope of the present study. However, the statistical approach used here ensures that the effects of NMS on respiratory and metabolic outcomes are not due to indirect effects via its influence on body mass. Considering that the manifestations of SA are highly diverse and likely reflect different trajectories and pathophysiological causes (Eckert, 2018), it is unrealistic to expect a single animal model to accurately reproduce all phenotypes. However, the present study convincingly shows that experiencing stress during a critical period of development alters the ageing trajectory of female rats in ways that predispose to SA and related comorbidities. Ultimately, other aspects such as cardiovascular function need to be addressed to fully appreciate the value of this model, but nonetheless the results reported here identify stress as an important factor in the aetiology of SA. This finding is an important step towards the development of new mechanistic questions and ultimately in the identification of factors to be considered in the detection and prevention of SA, especially in ageing women.

## AUTHOR CONTRIBUTIONS

Richard Kinkead and Danuzia Ambrozio‐Marques conceptualized and designed this study. Danuzia Ambrozio‐Marques, Loralie Mei Guay, Alicia A. Koogler, and Tim D. Ostrowski performed the experiments and analysed the data. Richard Kinkead and Danuzia Ambrozio‐Marques wrote the paper. All authors reviewed and edited the manuscript. All authors have read and approved the final version of this manuscript and agree to be accountable for all aspects of the work in ensuring that questions related to the accuracy or integrity of any part of the work are appropriately investigated and resolved. All persons designated as authors qualify for authorship, and all those who qualify for authorship are listed.

## CONFLICT OF INTEREST

None declared.

## Data Availability

All data supporting the results are reported in the manuscript. Raw results can be provided upon request.
